# Imaging the Sialome during Zebrafish Development with Copper-Free Click Chemistry

**DOI:** 10.1002/cbic.201100649

**Published:** 2012-01-20

**Authors:** Karen W Dehnert, Jeremy M Baskin, Scott T Laughlin, Brendan J Beahm, Natasha N Naidu, Sharon L Amacher, Carolyn R Bertozzi

**Affiliations:** a Department of Chemistry, University of CaliforniaB84 Hildebrand Hall 1460, Berkeley, CA 94720 (USA) E-mail: crb@berkeley.edu; b Glycotechnology Core Resource, University of CaliforniaSan Diego, La Jolla, CA 92093 (USA); c Department of Molecular and Cell Biology, University of CaliforniaBerkeley, CA 94720 (USA); d Howard Hughes Medical Institute, University of CaliforniaBerkeley, CA 94720 (USA)

**Keywords:** bioorthogonal chemistry, embryonic development, fluorescence imaging, glycosylation, sialic acids

All cells in vertebrates are adorned with sialylated glycoproteins and glycolipids that collectively comprise the sialome.^1^, ^2^ Because sialic acid residues often occupy terminal positions on these structures, they are well situated to control biological interactions at the cell surface. For example, on N- and O-glycans, sialic acids contribute to recognition sites for glycan-binding proteins and mask underlying epitopes such as terminal galactose residues that might otherwise dominate a glycoprotein's biology.^3^, ^4^ Linear polymers of sialic acid, which are called polysialic acid and found primarily as posttranslational modifications of the neural cell adhesion molecule, modulate cell–cell adhesion^5^, ^6^ and regulate neuronal migration and differentiation during development.^7^, ^8^ Sialic acids are also critical components of gangliosides, a family of glycolipids especially abundant in the brain but also found on all other vertebrate cells where they mediate cell–cell interactions and regulate the responsiveness of some signaling receptors.^9^ Indeed, the importance of sialic acids in development is demonstrated by the observation that disruption of de novo sialic acid biosynthesis in mice causes embryonic lethality.^10^ Efforts to understand the functions of sialic acids using traditional approaches have been stymied by genetic redundancy of sialyltransferase family members and a lack of tools for probing sialic acids at the biochemical level.^11^, ^12^ As a result, we and others have sought to study sialic acids in vivo by developing methods to image sialylated glycans directly within living systems.^13^–^17^

We previously found that sialylated glycans can be labeled in cultured cells and live mice by supplying the sialic acid biosynthetic pathway with azide-labeled analogues of the biosynthetic precursor *N*-acetylmannosamine (ManNAc).^18^–^20^ In this strategy, cells are treated with cell-permeable, peracetylated *N*-azidoacetylmannosamine (Ac_4_ManNAz), which is converted to the corresponding azido sialic acid (SiaNAz) and incorporated into cell-surface glycans (Figure 1 A).^21^, ^22^ The azidosugar can then be visualized using a copper-free click chemistry reaction with a difluorinated cyclooctyne (DIFO) reagent conjugated to an imaging probe.^23^

**Figure 1:**
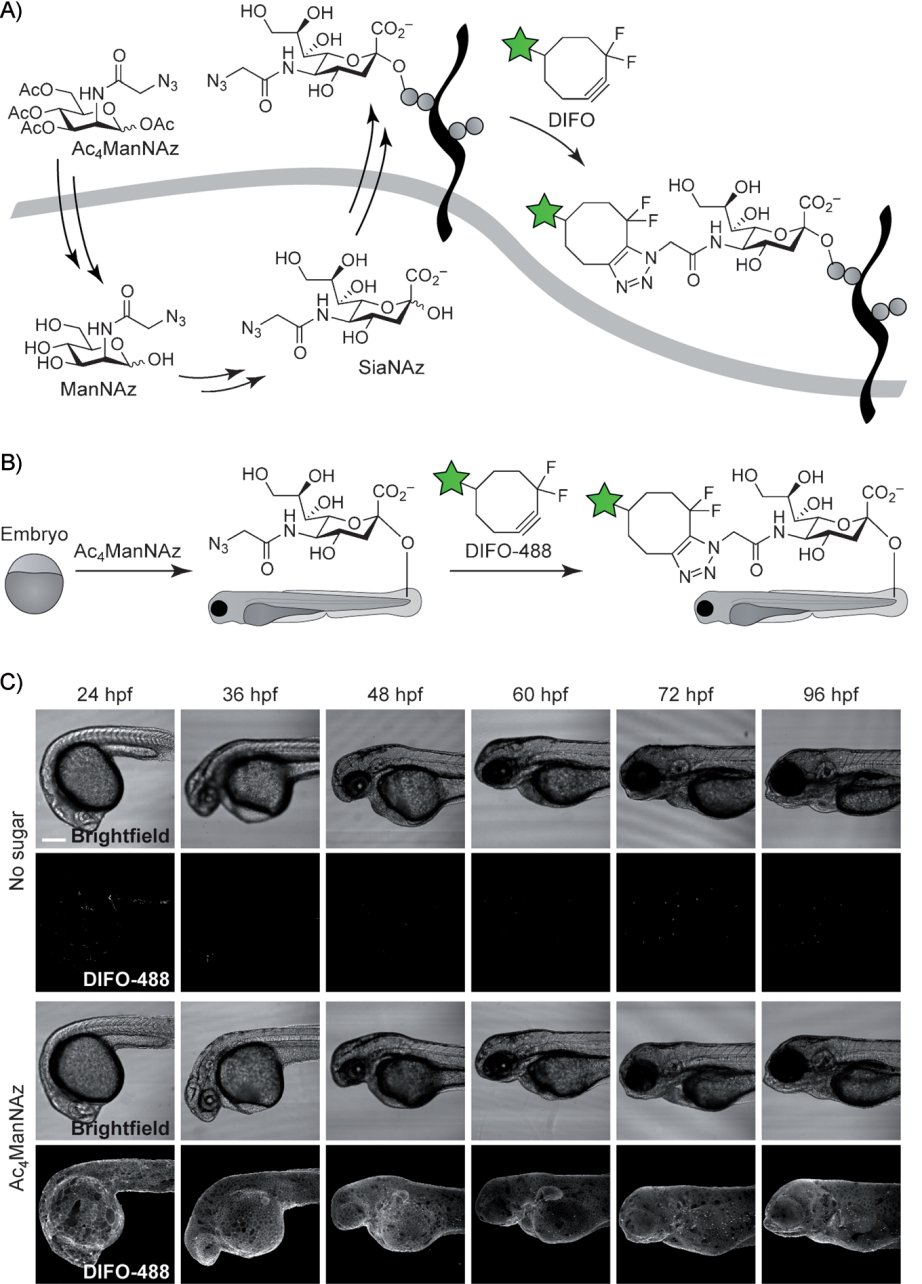
Metabolic labeling strategy and visualization of sialylated glycans in developing zebrafish. A) Scheme for metabolic labeling of sialylated glycans. Cell-permeable, peracetylated *N*-azidoacetylmannosamine (Ac_4_ManNAz) enters the cell, and once its acetyl groups are cleaved, ManNAz traverses the steps of the sialic acid biosynthetic pathway and is converted to azido sialic acid (SiaNAz). After conversion to the nucleotide sugar CMP-SiaNAz, SiaNAz is incorporated into cell-surface glycoconjugates by sialyltransferase enzymes. At the cell surface, SiaNAz is visualized by reaction with fluorophore-conjugated difluorinated cyclooctyne (DIFO) reagents. B) Scheme for visualizing sialylated glycans in zebrafish embryos. Embryos are incubated in solution containing Ac_4_ManNAz, which provides metabolic labeling of cell-surface sialylated glycans over the course of development. In a second step, reaction with DIFO-488 enables visualization of the labeled glycans. C) Treatment with Ac_4_ManNAz followed by reaction with DIFO-488 provides labeling of sialic acids in the enveloping layer during the first four days of development. Embryos were incubated in a solution containing Ac_4_ManNAz (or no sugar) beginning at 4 hpf and then were reacted with DIFO-488 at the indicated times. Shown are *Z* projection images of DIFO-488 fluorescence and corresponding brightfield images of embryos, viewed laterally. Scale bar: 200 μm.

We sought to extend this strategy toward imaging sialylated glycans in developing zebrafish. Zebrafish are a popular vertebrate model organism due in part to their well-characterized, external development^24^ and their transparent embryos, which are well suited for optical imaging.^25^ Surprisingly, little glycomics research has been performed using this attractive experimental system. Indeed, to our knowledge, global analysis of the sialome during zebrafish development has been limited to a single mass spectrometry-based report.^26^ We previously sought to image the sialome in developing zebrafish by applying a chemical technique, refined by Paulson and coworkers,^16^ in which aldehydes are installed on cell-surface sialic acids by oxidation with sodium periodate and then visualized by subsequent reaction with aminooxy-functionalized fluorescent probes.^27^ Although this technique enabled visualization of the steady-state sialome, metabolic labeling would provide an opportunity to monitor biosynthetic flux as new azide-labeled glycans traverse the secretory pathway and are displayed at the cell surface. The metabolic labeling method is particularly well suited for monitoring dynamic changes in the sialome in a spatiotemporally resolved manner. We previously employed similar strategies to image mucin-type O-glycans and fucosylated glycans in zebrafish.^27^–^29^ We now extend the metabolic labeling approach to image sialic acids in this model organism.

To label sialylated glycans in zebrafish over the first four days of development, we incubated embryos in medium containing Ac_4_ManNAz from 4 to 96 h post-fertilization (hpf; Figure 1 B). Then, at several stages of development, we reacted the embryos with an Alexa Fluor 488 conjugate of DIFO (DIFO-488) and imaged them by confocal microscopy. We observed a robust fluorescence signal in the enveloping layers of embryos from 24 to 96 hpf (Figure 1 C). Importantly, we observed little background fluorescence in embryos incubated in medium that did not contain Ac_4_ManNAz, indicating that the DIFO-488 signal that we observed was azide-specific. Additionally, the azidosugar and DIFO treatments did not result in any toxicity or developmental abnormalities in the treated embryos.

To temporally distinguish populations of sialoglycoconjugates, we performed two DIFO labeling reactions in succession, thereby marking sialic acids biosynthesized at different stages of development with distinct fluorophores (Figure S1 in the Supporting Information). Zebrafish embryos were incubated in medium containing Ac_4_ManNAz from 4 to 72 hpf and then were reacted with DIFO-488 to visualize the sialylated glycans that had been synthesized during the first three days of development. After this reaction, any remaining cell-surface azides were reduced to amines using tris(2-carboxyethyl)phosphine (TCEP), a mild reducing agent.

The embryos were then returned to Ac_4_ManNAz-containing medium and allowed to further develop from 73 to 76 hpf. During this time, azide-labeled precursor sugars continued to traverse intracellular biosynthetic pathways and become incorporated into newly-synthesized glycans. The SiaNAz-containing glycans presented at the cell surface during this period were then reacted with DIFO-555, at 76 hpf. Thus, glycans synthesized and trafficked to the cell surface between 73 and 76 hpf and subsequently labeled with DIFO-555 could be distinguished from the DIFO-488-labeled glycans that were synthesized during the first three days of development.

This method revealed several regions of the embryo in which areas of new glycosylation differed from old. Cells in the ventral jaw region of the embryo, for example, exhibited a corrugated pattern of labeling, with DIFO-488-labeled glycans concentrated in peaks that extended outward from the ventral surface; DIFO-555-labeled glycans were concentrated in troughs located more dorsally (Figure 2 A–C and Figure S2). In the olfactory organ, older glycans labeled by DIFO-488 were found in both apical and basal regions of the olfactory epithelium at the base of the olfactory pit, whereas newly synthesized, DIFO-555-labeled glycans appeared to be restricted to the apical regions of the epithelium and the periphery of the olfactory pit (Figure 2 D-(F).

**Figure 2:**
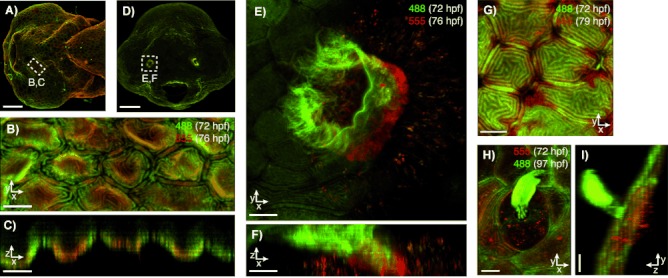
Two-color labeling differentiates sialylated glycans synthesized at different developmental stages. A–F) Embryos were incubated with Ac_4_ManNAz (5 mm) beginning at 4 hpf, then reacted with DIFO-488 (100 μm, 1 h) at 72 hpf. Following this reaction, the embryos were incubated with a solution of tris(2-carboxyethyl)phosphine (TCEP, 50 mm, 10 min) to quench any remaining cell-surface azides. The embryos were then incubated with additional Ac_4_ManNAz for 3 h before treatment with DIFO-555 (100 μm, 1 h) at 76 hpf. A) *Z* projection of DIFO-488 and DIFO-555 fluorescence from an embryo's ventral surface, with the area of interest for B and C indicated. B) *Z* projection fluorescence image of enveloping layer cells in the ventral jaw region. C) A single *x*–*z* plane from the middle of the region indicated in A and B, from an image linearly interpolated by a factor of 3 along the *z* axis. D) Frontal view of an embryo with the area of interest for E and F indicated. E) *Z* projection fluorescence image of the olfactory organ and surrounding epithelium. F) *Y* projection of the same region shown in D, from an image linearly interpolated by a factor of 3 along the *z* axis. G) After incubation from 4 to 72 hpf with Ac_4_ManNAz (5 mm), embryos were reacted first with DIFO-488 (100 μm, 1 h) at 72 hpf, then with TCEP (50 mm, 10 min), and then returned to Ac_4_ManNAz-containing medium for 6 h before treatment with DIFO-555 (100 μm, 1 h) at 79 hpf. Shown is a *Z* projection of DIFO-488 and DIFO-555 fluorescence of epithelial cells. H–I) Ac_4_ManNAz-treated embryos were reacted first with DIFO-555 (100 μm, 1 h) at 72 hpf, then with TCEP (50 mm, 10 min), and then returned to Ac_4_ManNAz-containing medium for 24 h and finally reacted with DIFO-488 (100 μm, 1 h) at 97 hpf. H) *Z* projection fluorescence image of a kinocilium from mechanosensory hair cells. I) *X* projection fluorescence image of the region shown in H, from an image linearly interpolated by a factor of 4 along the *z* axis. Green, DIFO-488; red, DIFO-555. Scale bars: 100 μm (A, D); 10 μm (B, C, E, F, G, H, I).

We then extended the time between the first and second reactions, first labeling with DIFO-488 at 72 hpf and then with DIFO-555 at 79 hpf. In most of the epithelium, we observed labeling by both DIFO-488 and DIFO-555, suggesting that sialylated glycans were expressed throughout the experiment, both before and after 72 hpf (Figure 2 G). However, portions of cells near the junctions with one another were labeled primarily with DIFO-555. Perhaps de novo glycan biosynthesis occurs at higher levels at these cell junctions, or perhaps those regions of the cells first became solvent-exposed during the 6 h time lapse, and so were not accessible to the DIFO-488 reagent during the first reaction.

Finally, we performed labeling experiments with a 24 h Ac_4_ManNAz incubation between the first and second DIFO reactions. For these experiments, embryos were labeled first with DIFO-555 at 72 hpf and then with DIFO-488 at 97 hpf. We observed particularly striking DIFO-488 labeling of the kinocilia of mechanosensory hair cells in the lateral line, indicating that the sialylation of these structures had occurred primarily between 72 and 97 hpf (Figure 2 H–I). Together, the results of these labeling experiments are similar to those observed with labeling of mucin-type O-glycans.^28^ Sialic acids are often found as terminal monosaccharides on O-glycans, and it is not surprising that these two partially overlapping classes of glycans have similar expression patterns during development.

Although incubation with Ac_4_ManNAz allowed us to image sialic acids from 24 hpf to 96 hpf, we did not observe any azide-specific signal in embryos younger than 24 hpf. To visualize glycans during early stages of embryonic development, we chose to provide embryos with a downstream metabolic intermediate. We have found previously that microinjection of embryos at the 1–8-cell stage with other azidosugars, including *N*-azidoacetylgalactosamine (GalNAz) and GDP-6-azidofucose (GDP-FucAz), results in labeling of mucin-type O-glycans and fucosylated glycans, respectively, well before 24 hpf.^27^, ^29^ To image sialic acids during early embryogenesis, we directly microinjected embryos with the metabolic intermediate SiaNAz and allowed them to develop, and then we reacted them with DIFO-488 at several different time points during the gastrulation and early segmentation periods. We observed cell-surface labeling of sialic acids at 8.5 hpf, during gastrulation (Figure 3 A). Notably, we did not observe fluorescence signal in the yolk, a phenomenon that had occurred with sodium periodate treatment at all developmental time points prior to the end of gastrulation.^27^ We also observed a robust fluorescence signal in the enveloping layer of embryos at 12 hpf, during the early segmentation period of development (Figure 3 B).

**Figure 3:**
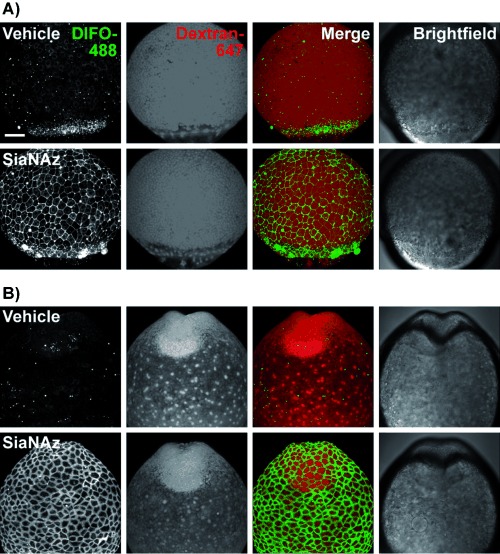
Microinjection of SiaNAz provides labeling of sialylated glycans during gastrulation and early segmentation periods. Embryos were microinjected with vehicle containing a tracer dye, Alexa Fluor 647-conjugated dextran, either alone (rows 1 and 3) or with 50 pmol of SiaNAz (rows 2 and 4). The embryos were allowed to develop and then were reacted with DIFO-488 (200 μm, 40 min (A) or 30 min (B)) and imaged either at 8.5 hpf (A) or 12 hpf (B). Shown are *Z* projection images of DIFO-488 and dextran-647 fluorescence and corresponding brightfield images. Scale bar: 100 μm.

We allowed the SiaNAz-injected embryos to continue to develop over the next four days. On each day, we reacted several embryos with DIFO-488 and imaged them by confocal microscopy. We observed fluorescence in the enveloping layer of embryos injected with SiaNAz and no background fluorescence in embryos injected with vehicle alone on each day of the four-day experiment (Figure S3). As with Ac_4_ManNAz treatment, we observed no developmental abnormalities in SiaNAz-injected embryos, further demonstrating that SiaNAz metabolism and the reagents utilized for copper-free click chemistry are not toxic to zebrafish embryos.

Given the importance of sialic acid in vertebrate development, the observed lack of embryonic abnormalities in Ac_4_ManNAz-treated embryos might seem surprising. However, replacement of wild-type sialic acids with SiaNAz is a far less perturbing modification than a global knockout. Additionally, only a fraction of wild-type sialic acid residues are likely replaced with SiaNAz. To quantify this number, we determined the ratio of wild-type sialic acid to SiaNAz residues in labeled zebrafish lysates by using an HPLC assay.^22^ Embryos were microinjected with SiaNAz or vehicle alone and then were allowed to develop and incorporate SiaNAz into cellular glycoproteins over 24 h. The entire proteome was isolated, and the incorporated sialic acids were released by mild acid treatment and derivatized with 1,2-diamino-4,5-methylenedioxybenzene (DMB). The derivatized sialic acids were then identified and quantified by reversed-phase HPLC. Using this assay, we found that zebrafish glycoproteins contained both *N*-glycolylneuraminic acid (Neu5 Gc) and *N*-acetylneuraminic acid (Neu5 Ac), as expected, and zebrafish injected with vehicle alone did not yield any SiaNAz peak (Figure 4). However, in SiaNAz-injected zebrafish, between 7 and 18 % of sialic acids in glycoproteins contained SiaNAz instead of one of the naturally occurring sialic acids (Table S1). These values, which varied somewhat depending on the sample batch, reflect the average of all cell types in the embryo, but it is likely that some tissues, such as the enveloping layer, are more highly labeled than others. Nevertheless, this extent of SiaNAz incorporation is apparently sufficient for imaging purposes without interfering with the normal biology of the organism.

**Figure 4:**
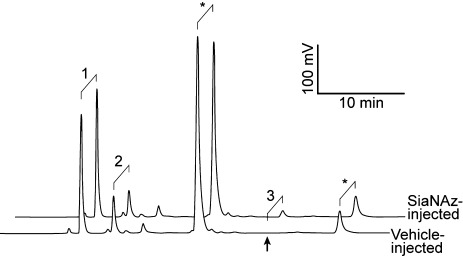
SiaNAz is incorporated into zebrafish glycoproteins. Proteins from embryos injected with SiaNAz or vehicle alone were isolated from lysate, and sialic acids were released, derivatized with DMB, and analyzed by HPLC. Peaks were identified by a separate injection of known standards: peak 1, *N*-glycolylneuraminic acid; peak 2, *N*-acetylneuraminic acid; peak 3, SiaNAz. Starred peaks correspond to contaminants that were also observed in blank injections.

Several conclusions emerge from this work. First, metabolic labeling and copper-free click chemistry revealed that cell-surface sialosides are biosynthesized in zebrafish embryos as early as 8.5 hpf. Additionally, dual-labeling experiments showed that the sialome is spatially and temporally regulated during development, with areas of particularly robust new sialoglycoconjugate expression in the olfactory organ and on the kinocilia of mechanosensory hair cells at different stages of development. Although this platform gives no structural detail regarding the linkages and scaffolds of labeled sialic acid residues, it can be employed to probe global dynamics of the sialome in live organisms and in real time. In this regard, imaging by metabolic labeling is a powerful complement to mass spectrometry-based profiling approaches, which provide structural details but without a spatiotemporal context. An interesting future direction might be to combine metabolic labeling with mass spectrometry-based proteomics.

## Experimental Section

Experimental procedures are described in the Supporting Information. Experiments involving live zebrafish were approved by the UC Berkeley Animal Care and Use Committee under Animal Use Protocol #R255.

## Supplementary material

Detailed facts of importance to specialist readers are published as ”Supporting Information”. Such documents are peer-reviewed, but not copy-edited or typeset. They are made available as submitted by the authors.
