# Supplementary material for: Imaging the Sialome during Zebrafish Development with Copper-Free Click Chemistry
Source: Chembiochem. 2012 Jan 20;13(3):353–7. doi: 10.1002/cbic.201100649 (PMC3385855; doi:10.1002/cbic.201100649)
Supplement: Supplementary file 1 [file cbic0013-0353-SD1.pdf]

CHEMBIOCHEM

Supporting Information

© Copyright Wiley-VCH Verlag GmbH & Co. KGaA, 69451 Weinheim, 2012

Imaging the Sialome during Zebrafish Development with Copper-Free Click Chemistry

Karen W. Dehnert,[a] Jeremy M. Baskin,[a] Scott T. Laughlin,[a] Brendan J. Beahm,[a] Natasha N. Naidu,[b]
Sharon L. Amacher,[c] and Carolyn R. Bertozzi*[a, c, d]

cbic_201100649_sm_miscellaneous_information.pdf

Experimental Section

General materials and methods. Chemical reagents were obtained from the commercial suppliers indicated and were used without further purification. Pronase (protease, Type XIV, from *Streptomyces griseus*), *N*-phenylthiourea (PTU), tricaine (ethyl 3-aminobenzoate methanesulfonate), and tris(2-carboxyethyl)phosphine (TCEP) hydrochloride were obtained from Sigma-Aldrich. Tetramethylrhodamine-conjugated dextran (dextran, tetramethylrhodamine, 10,000 MW, lysine fixable) and Alexa Fluor 647-conjugated dextran (dextran, Alexa Fluor 647, 10,000 MW, anionic, lysine fixable) were obtained from Invitrogen. Complete, mini, EDTA-free protease inhibitor cocktail tablets were obtained from Roche. $Ac_4ManNAz$,[1] SiaNAz,[2] DIFO-488,[3] and DIFO-555[4] were prepared as described previously. Confocal microscopy was performed at the UC Berkeley Molecular Imaging Center.

Zebrafish lines and husbandry. AB-derived (wild-type) adult zebrafish were kept at 28 °C on a 14-h light/10-h dark cycle. Embryos were obtained from natural spawnings and raised at 28.5 °C in embryo medium (150 mM NaCl, 0.5 mM KCl, 1.0 mM $CaCl_2$, 0.37 mM KH_2PO_4, 0.05 mM Na_2HPO_4, 2.0 mM $MgSO_4$, 0.71 mM $NaHCO_3$ in deionized H_2O, pH 7.4). The embryos and larvae were staged developmentally according to Kimmel *et al.*[5] Experiments involving zebrafish were approved by the UC Berkeley Animal Care and Use Committee under Animal Use Protocol #R255.

Labeling of sialic acids with $Ac_4ManNAz$ and DIFO-488. Zebrafish embryos were dechorionated with pronase (1 mg/mL in embryo medium) at 4 hours post-fertilization (hpf). The embryos were incubated in embryo medium containing 5 mM $Ac_4ManNAz$ over the

course of development. PTU (131 μM) was included in the medium beginning at 12 hpf to inhibit melanin production. At each time indicated (24, 36, 48, 72, and 96 hpf), several embryos were removed from the $Ac_4ManNAz$-containing solution, rinsed, and incubated in embryo medium containing DIFO-488 (100 μM) for 1 h at 28.5 °C. The embryos were then rinsed by sequential transfers through six 25-cm tissue culture dishes. The embryos were anesthetized with tricaine (2.6 μM in embryo medium) and mounted between two cover slips in embryo medium containing 0.6% low melting point agarose, 2.6 μM tricaine, and 131 μM PTU.

Two-color labeling of sialylated glycans. Embryos were dechorionated and incubated with $Ac_4ManNAz$ as described above. At 72 hpf, the embryos were removed from $Ac_4ManNAz$-containing medium and rinsed. For the images shown in Figure 2A–G, the embryos were incubated in embryo medium containing DIFO-488 (100 μM) for 1 h at 28.5 °C. The embryos were rinsed and then incubated in embryo medium containing TCEP (50 mM, pH 7.4) for 10 min. The embryos were rinsed six more times and then returned to $Ac_4ManNAz$-containing medium. After an additional 3 h or 6 h incubation with $Ac_4ManNAz$ (at 76 hpf, for Figures 2A–F, or 79 hpf, for Figure 2G), the embryos were reacted with DIFO-555 (100 μM) for 1 h at 28.5 °C. Alternatively, for the images shown in Figure 2H–I, the embryos were incubated first with DIFO-555 at 72 hpf, then reacted with TCEP and returned to $Ac_4ManNAz$-containing medium for an additional 24 h before a second reaction with DIFO-488 at 97 hpf. After both reactions, the embryos were rinsed by sequential transfers through six 25-cm tissue culture dishes, anesthetized with tricaine (2.6 μM in embryo medium), and mounted between two cover slips in embryo medium containing 0.6% low melting point agarose, 2.6 μM tricaine, and 131 μM PTU.

Microinjection of SiaNAz and visualization of sialylated glycans with DIFO-488. For the images shown in Figure 3, embryos were microinjected into the yolk at the 1–4-cell stage with 2 nL of either vehicle alone (2% dextran-647 and 0.2 M KCl in deionized water) or vehicle containing 25 mM SiaNAz. For the images shown in Figure S3 in the Supporting Information, the embryos were microinjected into the yolk at the 1–4-cell stage with 4 nL of either vehicle alone (5% tetramethylrhodamine-dextran and 0.2 M KCl in deionized water) or vehicle containing 25 mM SiaNAz. The embryos were dechorionated with pronase at 4 hpf as described and allowed to continue to develop at 28.5 °C in agarose-coated dishes containing embryo medium. For the images shown in Figure 3, embryos at 7 hpf (Figure 3A) or 11 hpf (Figure 3B) were incubated in embryo medium containing DIFO-488 (200 μM) for 40 min (Figure 3A) or 30 min (Figure 3B) at 28.5 °C. For the images shown in Figure S3, the embryos were raised in medium containing PTU (131 μM), and at the times indicated (24, 48, 72, and 96 hpf), they were incubated in embryo medium containing DIFO-488 (100 μM) for 1 h at 28.5 °C.

After the copper-free click chemistry reactions, the embryos were rinsed by sequential transfers through six 10-cm agarose-coated dishes. The embryos were mounted between two cover slips in 0.6% low melting point agarose in embryo medium for imaging. Embryos 24 hpf and older were first anesthetized with tricaine (2.6 μM) and were mounted in agarose-containing medium with 2.6 μM tricaine and 131 μM PTU.

Imaging of glycans by confocal microscopy. Fluorescence and brightfield images shown in Figures 1, 2, and S3 were acquired on a Zeiss LSM 510 META laser scanning confocal microscope equipped with 488, 543, and 633 nm laser lines and the following objectives: 10x/0.30NA Plan-Neofluar air objective, 20x/0.50NA Achroplan water dipping objective, 40x/0.80NA Achroplan IR water dipping objective, and 63x/0.95NA Achroplan IR water

dipping objective. Images shown in Figures 3 and S2 were obtained on a Zeiss LSM 780 AxioExaminer laser scanning confocal microscope equipped with 488 and 635 nm laser lines and a 20x/1.0NA Plan-Apochromat water dipping objective. All images were analyzed using Slidebook 5.0 (Intelligent Imaging Innovations). Images shown as x- or y-projections were linearly interpolated along the z axis using Slidebook's interpolation function. For the images shown in Figure 2B–C, the original voxel size was 0.17 μm in the x and y directions and 0.50 μm in the z direction, and the image was interpolated along the z-axis by a factor of 3 in Figure 2C. For the images shown in Figure 2E–F, the original voxel size was 0.15 μm in the x and y directions and 0.50 μm in the z direction, and the image was interpolated along the z-axis by a factor of 3 in Figure 2F. For the images shown in Figure 2H–I, the original voxel size was 0.58 μm in the x and y directions and 2.50 μm in the z direction, and the image was interpolated along the z-axis by a factor of 4 for Figure 2I.

Quantification of SiaNAz incorporation in zebrafish lysates by DMB functionalization and HPLC detection. Embryos were microinjected into the yolk at the 1–4-cell stage with 2 nL of either vehicle alone (2% dextran-647 and 0.2 M KCl in deionized water) or vehicle containing 25 mM SiaNAz. The embryos were batch dechorionated at 4 hpf using pronase (1 mg/mL in embryo medium), then allowed to continue to develop at 28.5 °C. At 24 hpf, the embryos were rinsed once with embryo medium and treated with tricaine. The embryos were then deyolked in samples containing 25 embryos each using a protocol modified from Link and coworkers.[6] In this procedure, the embryos were rinsed once with deyolking buffer (55 mM NaCl, 1.8 mM KCl, 1.25 mM $NaHCO_3$, 2.7 mM $CaCl_2$) and then added to a microcentrifuge tube containing 1 mL deyolking buffer. The embryos were deyolked by pipetting up and down with a P200 tip. The samples were shaken (1100 rpm, 3 min), then centrifuged (340 x *g*, 2 min) to pellet the cells, and the supernatant was removed. The cells

were washed twice by adding 1 mL wash buffer (110 mM NaCl, 3.5 mM KCl, 2.7 mM $CaCl_2$,10 mM Tris/Cl, pH 8.5) to the samples, shaking (1100 rpm, 3 min), centrifuging (340 x *g*, 2 min), and then removing the supernatant and repeating once more. After the two washes, to each sample was added 500 μL lysis buffer (150 mM NaCl, 20 mM Tris, 1% Nonidet P 40 Substitute, pH 7.4, with one Roche complete, mini, EDTA-free protease inhibitor cocktail tablet for 10 mL of lysis buffer). The samples were sonicated at 4 °C using a microtip and two 30-s, ~3-W pulses. The samples were then centrifuged (13000 rpm, 4 °C, 10 min) to remove insoluble debris, and the supernatant was collected.

To precipitate the protein, acetone (4.5 mL) was added to each sample to result in an acetone:water ratio of 9:1. The samples were incubated at -80 °C for 24 h. The samples were then centrifuged (3600 x *g*, 4 °C, 30 min), and the supernatant was removed. To the pellet was added methanol (5 mL), and the pellet was disrupted by vortexing and sonication. The samples were then incubated at -80 °C for 48 h, centrifuged again (3600 x *g*, 4 °C, 30 min), and the methanol was removed. This methanol wash procedure was repeated once more.

To release sialic acids from the proteins, the samples were treated with acetic acid (2 M) at 80 °C for 3 h. The released sialic acids were collected through a 3000 Da molecular weight cut-off filter and then modified with 1,2-diamino-4,5-methylenedioxybenzene (DMB) by treating with a solution containing DMB (13 mM), acetic acid (1.4 M), β-mercaptoethanol (750 mM), and sodium dithionite (18 mM) at 50 °C for 2.5 h in the dark. One-fifth of the sample was then injected onto a Dionex C18 reverse-phase liquid chromatography column (5 μm, 4.6 mm x 250 mm) with a mobile phase containing 7% methanol and a gradient of 7 to 11% acetonitrile (86 to 82% water) over 40 min. Modified sialic acids were monitored by fluorescence with excitation at 373 nm and emission detection at 448 nm.

References

[1] E. Saxon, C. R. Bertozzi, *Science* **2000**, *287*, 2007-2010.
[2] H. Yu, H. Yu, R. Karpel, X. Chen, *Bioorg. Med. Chem.* **2004**, *12*, 6427-6435.
[3] J. M. Baskin, J. A. Prescher, S. T. Laughlin, N. J. Agard, P. V. Chang, I. A. Miller, A. Lo, J. A. Codelli, C. R. Bertozzi, *Proc. Natl. Acad. Sci. U.S.A.* **2007**, *104*, 16793-16797.
[4] S. T. Laughlin, J. M. Baskin, S. L. Amacher, C. R. Bertozzi, *Science* **2008**, *320*, 664-667.
[5] C. B. Kimmel, W. W. Ballard, S. R. Kimmel, B. Ullmann, T. F. Schilling, *Dev. Dyn.* **1995**, *203*, 253-310.
[6] V. Link, A. Shevchenko, C. P. Heisenberg, *BMC Dev. Biol.* **2006**, *6*, 1.

Supporting Table

Table S1. Sialic acid species detected in lysates from vehicle- and SiaNAz-injected embryos. Lysates were prepared from 24-hpf embryos that were had been injected at the 1–4-cell stage with either vehicle alone or vehicle containing 50 pmol SiaNAz. Proteins were precipitated using acetone and washed twice with cold methanol, and sialic acids were released using acetic acid, derivatized with DMB, and analyzed by HPLC. The values shown are the percent of each individual sialic acid species as a percentage of the total sialic acids detected. Duplicate measurements are shown for vehicle-injected samples, and triplicate measurements are shown for SiaNAz-injected samples.

Sample	Neu5Gc	Neu5Ac	SiaNAz
Vehicle-injected	78, 72	22, 28	–, –
SiaNAz-injected	80, 61, 67	13, 23, 15	7, 16, 18

Values indicate percent of total sialic acids

Supporting Figures

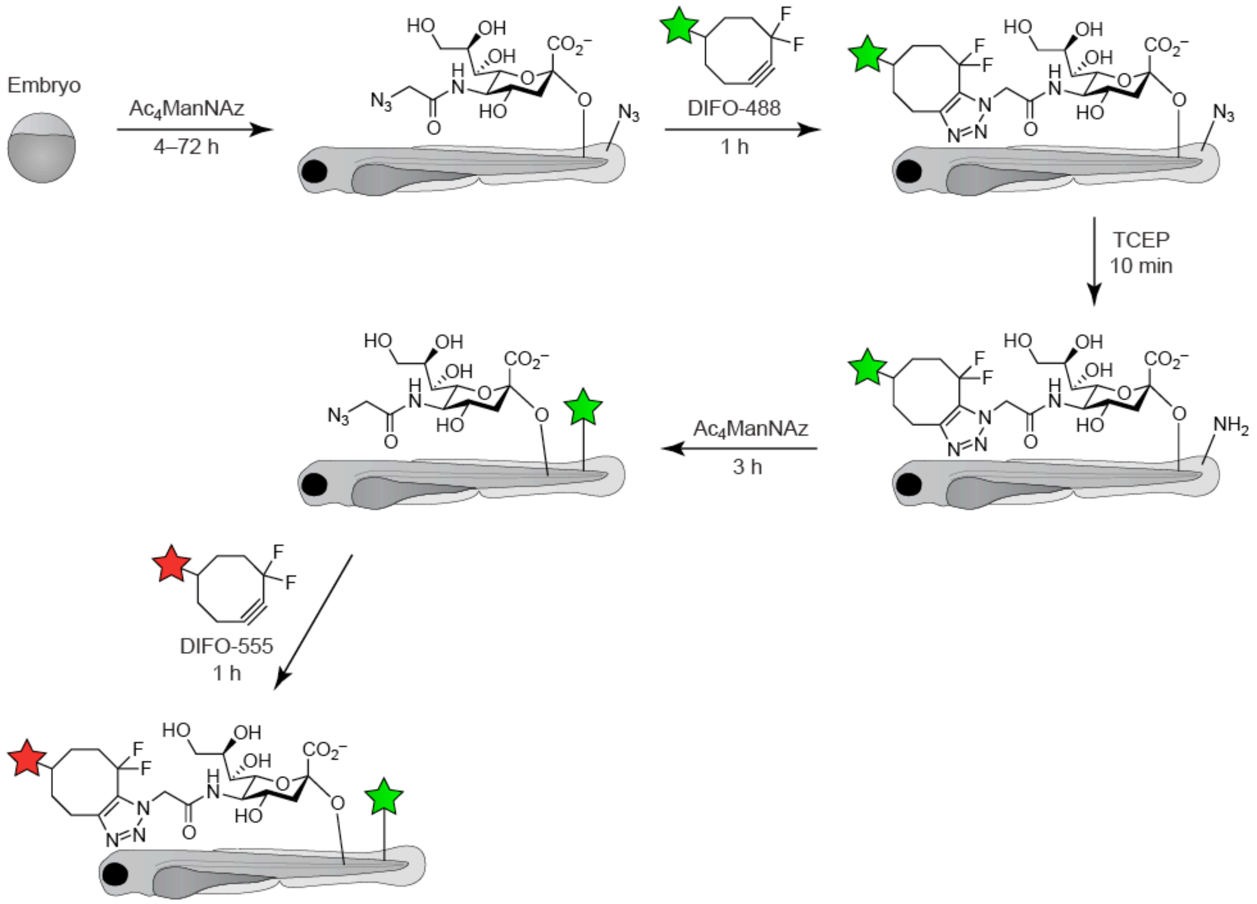

Figure S1. Strategy for two-color labeling of *de novo* sialic acid biosynthesis. Embryos are incubated with $Ac_4ManNAz$ from 4 to 72 hpf and then reacted with DIFO-488. Unreacted cell-surface azides are reduced to amines by treatment with tris(2-carboxyethyl)phosphine (TCEP) and the embryos are returned to medium containing $Ac_4ManNAz$ for further metabolic labeling. Finally, newly-synthesized SiaNAz-containing glycans are reacted with DIFO-555.

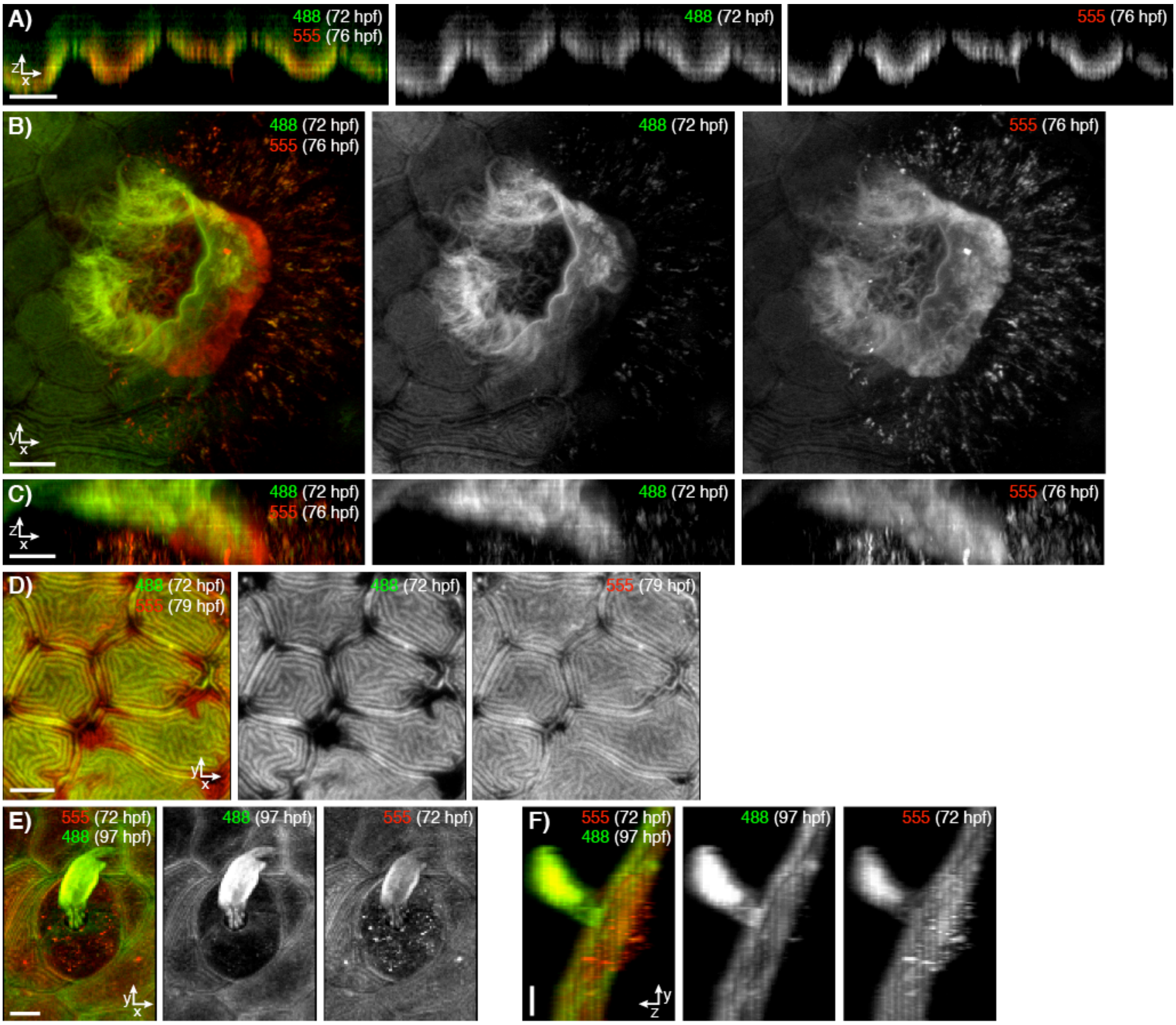

Figure S2. Two-color labeling with DIFO-488 and DIFO-555 differentiates sialylated glycans synthesized at different stages of development. Single-channel images alongside some of the merged images shown in Figure 2 of the main text. For these experiments, embryos were incubated with $Ac_4ManNAz$ (5 mM) from 4 to 72 hpf. A–C) Embryos were reacted with DIFO-488 (100 μM, 1 h) at 72 hpf, then incubated with additional $Ac_4ManNAz$ for 3 h before treatment with DIFO-555 (100 μM, 1 h) at 76 hpf. A) A single x-z plane from the jaw region, from an image linearly interpolated by a factor of 3 along the z-axis. B) Z-projection fluorescence image of the olfactory organ and surrounding epithelium. C) Y-projection of the same region shown in B, from an image linearly interpolated by a factor of 3 along the z-axis. D) Embryos were reacted first with DIFO-488 (100 μM, 1 h) at 72 hpf, then returned to $Ac_4ManNAz$-containing medium for 6 h and finally reacted with DIFO-555 (100 μM, 1 h) at 79 hpf. Shown is a z-projection image of DIFO-488 and DIFO-555 fluorescence of epithelial cells. E–F) Embryos were first reacted with DIFO-555 (100 μM, 1 h) at 72 hpf, then returned to $Ac_4ManNAz$-containing medium for 24 h and reacted with DIFO-488 (100 μM, 1 h) at 97 hpf. E) Z-projection fluorescence image of a kinocilium from mechanosensory hair cells. F) X-projection fluorescence image of the region shown in E, from an image linearly interpolated by a factor of 4 along the z-axis. Green, DIFO-488; red, DIFO-555. Scale bars: 10 μm.

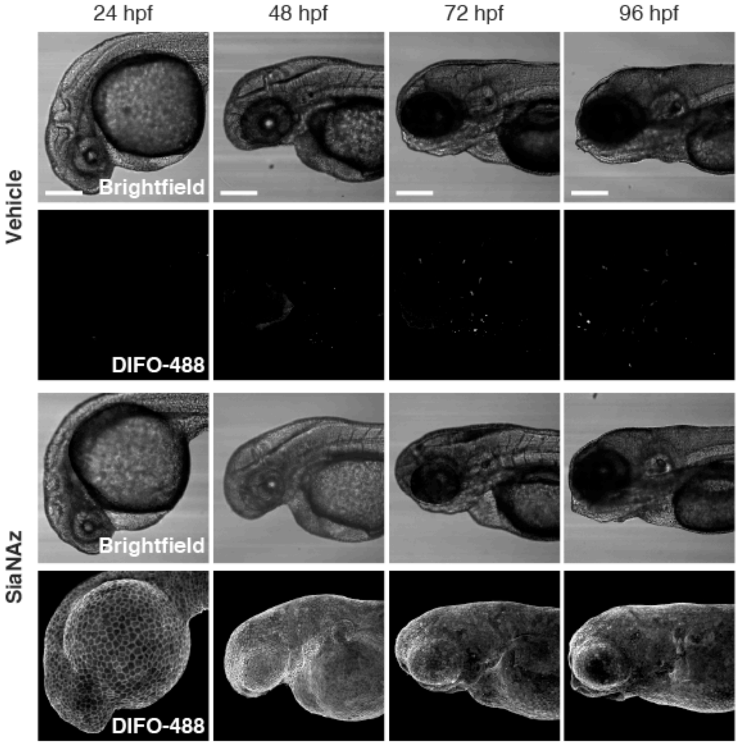

Figure S3. Microinjection of SiaNAz provides labeling of sialylated glycans during the first 96 h of development. Embryos were microinjected with SiaNAz or vehicle alone, allowed to develop to the developmental stages shown, and then reacted with DIFO-488. Shown are z-projection images of DIFO-488 fluorescence and corresponding brightfield images. Scale bars: 200 μm.
